# Protective Effects of Nucleobinding-2 After Cerebral Ischemia Via Modulating Bcl-2/Bax Ratio and Reducing Glial Fibrillary Acid Protein Expression

**DOI:** 10.32598/bcn.10.5.451

**Published:** 2019-09-01

**Authors:** Sohaila Erfani, Ali Moghimi, Nahid Aboutaleb, Mehdi Khaksari

**Affiliations:** 1. Department of Biology, Faculty of Science, Ferdowsi University of Mashhad, Mashhad, Iran.; 2. Department of Physiology, Faculty of Medicine, Physiology Research Center, Iran University of Medical Sciences, Tehran, Iran.; 3. Addiction Research Center, Shahroud University of Medical Sciences, Shahroud, Iran.

**Keywords:** Nucleobinding-2 (NUCB2), Nesfatin-1, Apoptosis, Astrogliosis, Hippocampus, Ischemia

## Abstract

**Introduction:**

Nucleobinding-2 (NUCB2) or nesfatin-1, a newly identified anorexigenic peptide, has antioxidant, anti-inflammatory, and anti-apoptotic properties. Brain ischemiareperfusion induces irreversible damages, especially in the hippocampus area. However, the therapeutic effects of NUCB2 have not been well investigated in cerebral ischemia. This study was designed for the first time to investigate the protective effects of NUCB2/Nesfatin-1 on the expression of apoptosis-related proteins and reactive astrogliosis level in the CA1 area of hippocampus in an experimental model of transient global cerebral ischemia.

**Methods:**

The male Wistar rats were randomly allocated into 4 groups (sham, NUCB2, ischemia-reperfusion, and ischemia-reperfusion+NUCB21) (n =7). The model of cerebral ischemia was prepared by common carotid arteries occlusion for 20 minutes. Nesfatin-1 (20 μg/kg) and saline (as a vehicle) were injected (intraperitoneally) at the beginning of the reperfusion period. The assessment of the protein expression levels was performed by immunofluorescence and immunohistochemical staining.

**Results:**

NUCB2 significantly reduced the Bax and GFAP protein levels in the CA1 area after ischemia (P<0.05). Also, NUCB2 increased Bcl-2 protein level (P<0.05). NUCB2 exerted protective effects against ischemic injury by the inhibition of astrocytes activation as an inflammatory response and decreased neuronal cell apoptosis.

**Conclusion:**

The present study provides the possible neuroprotective view of nesfatin-1 in the treatment of ischemia injury model in rat hippocampus.

## Highlights

- Nesfatin-1 treatment significantly reduced the Bax expression induced by cerebral ischemia.
- Nesfatin-1 treatment increased Bcl-2 protein level after cerebral ischemia.
- Nesfatin-1 treatment decreased glial fibrillary acid protein level after cerebral ischemia.

## Plain Language Summary

Cerebral ischemia-reperfusion (I/R) produces adverse pathological conditions. During ischemia, the blood supply to an organ decreases. The returned blood during reperfusion transfer oxygen to the cells that may damage the protein, DNA, and membrane of the plasma cells. Nesfatin-1 is the amino-terminal fragment of nucleobinding-2 (NUCB2) peptide, which is identified as an anorexigenic factor in the hypothalamus. Also, anti-apoptotic and anti-inflammatory properties of nesfatin-1 have been recently reported in subarachnoid hemorrhage and traumatic brain injury in rats. In this study, we tried to find out the effect of nesfatin-1 on apoptosis and astrogliosis after cerebral ischemia. The transient global cerebral I/R injury model was induced in 4 groups. Seven days after ischemia, immunofluorescence and immunohistochemical staining were used for identifying Bax and or Bcl-2 and glial fibrillary acid protein (GFAP) activation. The result demonstrated that nesfatin-1 might suppress apoptosis and neuroinflammation I/R via reduced Bax and glial fibrillary acid protein expression.

## 1. Introduction

Nesfatin-1 is the amino-terminal fragment of nucleobinding-2 (NUCB2) peptide, which is identified as an anorexigenic factor in the hypothalamus; it also has a wide distribution in the central nervous system (Kolgazi et al., 2015). It was shown that the neurons of the central nesfatinergic system that express nesfatin-1 respond to peripheral inflammatory signals, and it may have a coordinating role of nesfatinergic system for response to infection or inflammation (Bonnet et al., 2009). Besides, anti-apoptotic and anti-inflammatory properties of nesfatin-1 have been recently reported in model subarachnoid hemorrhage and traumatic brain injury in rats that were created by the diminution of caspase-3 activity and pro-inflammatory cytokines secretion (Özsavcí et al., 2011, Tang et al., 2012).

Ischemic stroke and the following reperfusion can happen after acute therapeutic intervention by creating irreversible brain damages, the leading cause of disability, and the second leading cause of mortality in the developed countries (Shi et al., 2017). Numerous recent studies have been conducted, in which the development of many neuroprotective treatment strategies can reduce brain damage following cerebral ischemia in animal models (Majid, 2014). It seems that the possible neuroprotective mechanisms inhibit the local inflammation, excitotoxicity, free radical damage, neuronal apoptosis, and calcium influx into cells, leading to both improving functional outcomes and decreasing infarct size (Tuttolomondo et al., 2015).

The CA1 neurons of the hippocampus are selectively vulnerable to transient global cerebral ischemia and delayed cell death occurring in this area of the brain a few days after reperfusion (Nishijima et al., 2015). It has been well demonstrated that in ischemic stroke, apoptosis can be inhibited by decreasing the proapoptotic proteins (cleaved caspase-3, caspase-9, and Bax) expression and increasing the antiapoptotic protein (i.e., Bcl-2) (Aboutaleb et al., 2015, Zheng et al., 2017). In addition, the survival of CA1 pyramidal neurons after transient global ischemia was enhanced in transgenic mice with overexpressing Bcl-2 protein (MacManus & Linnik, 1997).

The neuroinflammation responses that occur by immune mediators following brain ischemia have a significant role in creating neuronal cell death. Astrocytes are the essential mediators of the brain that have been reported to release various pro-inflammatory factors after ischemic injuries such as intermediate-filament and Glial Fibrillary Acid Protein (GFAP) (Kim, Park, Chang, Kim, & Lee, 2016). In mice without GFAP, the susceptibility to brain damage increases. Furthermore, the previous evidence has shown that reactive astrocytes up-regulate the intermediate-filament and GFAP in many neurodegenerative conditions such as ischemia. Thus, it was extensively applied as an alternative marker of neuronal injury in brain ischemia (Cordeau Jr, Lalancette-Hébert, Weng, & Kriz, 2008).

However, the role of NUCB2 or so-called nesfatin-1 in cerebral ischemia-reperfusion has been poorly investigated. Given the protective effects of nesfatin-1 against ischemia, we investigated the effects of nesfatin-1 administration on several GFAP-positive cells and apoptotic-related proteins (Bax/Bcl-2), following transient global cerebral ischemia-reperfusion.

## 2. Methods

### 2.1. Animals and drugs

The male Wistar rats (250–300 g) were placed in standard cages with controlled room temperature (22° C–24° C), humidity (45%–50%), and 12:12 h light-dark cycle. All animals had free access to food and water. All experiments and animal handling were accomplished according to the Helsinki Declaration and Ferdowsi University of Mashhad Ethics Committee guidelines. Nesfatin-1 (Sigma-Aldrich, Germany) was stored at −20° C and dissolved in saline when injection.

### 2.2. Experimental design and protocols

Immunofluorescence and immunohistochemical staining were done 7 days after cerebral ischemia induction in 4 groups; sham (n=7), ischemia-reperfusion (n=7), ischemia-reperfusion plus nesfatin-1 (n=7) and nesfatin-1 (n=7). The last two groups intraperitoneally received nesfatin-1 (20 μg/kg) at the beginning of reperfusion. Animals in the sham group were operated under the same surgical procedures, except that their common carotid arteries were not obstructed. Animals in the nesfatin-1 group did not undergo the operation for the induction of ischemia. Seven days after ischemia induction, all rats were anesthetized and undergone transcardiac perfusion for staining.

### 2.3. The model of transient global cerebral ischemia

The model of transient global cerebral ischemia was induced with Shamsaei et al. (Shamsaei, Khaksari, Erfani, Rajabi, & Aboutaleb, 2015) procedure. The rats were anesthetized (ketamine/xylazine, 40 mg/kg, IP) and underwent ischemia model operation.First, both common carotid arteries of the rats were carefully exposed and separated from their carotid sheet and vagus nerves. Then, both common carotid arteries were occluded using Yashargil Aneurysm micro-clips for 20 minutes. At the end of the occlusion period, the clips were expelled for quick reperfusion, and reflow of the blood was visually affirmed. The feedback-controlled warming system kept their rectal temperature at 36.5°c ± 0.5°c during the experimental time. After the operation, the rats were put in home confines with free access to food and water and kept independently for 7 days. At the start of the reperfusion, nesfatin-1 (20 μg/kg) was dissolved in saline and was infused intraperitoneally.

### 2.4. Tissue preparation for staining

Seven days after ischemia, the animals were deeply anesthetized with ketamine, and transcardiac perfusion was performed with 0.9% saline, followed by 4% paraformaldehyde in 0.1 M phosphate buffer (pH=7.4). Afterward, their brains were expelled and fixed in a similar fixative for 3 days interims and embedded in paraffin. Then, 7-μm coronal sections, as indicated by the Paxinos atlas (somewhere in the range of 3.3 mm and 4.2 mm back to bregma) were cut by a microtome for various staining techniques (Erfani et al., 2015a).

### 2.5. The measurement of Bcl-2 and Bax immunoreactivity and GFAP Immunohistochemical staining

Immunofluorescence staining was utilized for distinguishing Bcl-2 and Bax activation and immunohistochemical staining for GFAP activation, identifying tissue sections. The sections were incubated at 62°c for 20 minutes, rehydrated through a descending alcohol series, and treated with 10% hydrogen peroxide in methanol for 10 minutes to reduce endogenous peroxidase activity. After being washed in Tris buffer (pH=7.4), antigens were recovered via autoclaving for 11 minutes in citrate buffer (pH=6). After washing, the sections were blocked with 10% typical goat serum for 60 minutes. The sections were then incubated with anti- Bcl-2 and -Bax antibody (rabbit antibody against rat, Abcam, UK) as a primary antibody at 4°c temperature overnight. After washing in Phosphate-Buffered Saline (PBS), the sections were incubated with anti-rabbit secondary antibody conjugated with a fluorochrome (Abcam, UK) for 2 hours in the dark to accomplish visualization of the antigen. At that point, the sections were counterstained with 4′; 6-diamidino-2-phenylindole (DAPI) in PBS for 5 minutes for labeling the nucleus. Also, for GFAP, polyclonal secondary antibody (HRP) (Abcam, UK) was incubated for 30 minutes at room temperature by adding 3,3′-diaminobenzidine (DAB, Sigma, USA) to detect the antigen. Finally, counterstaining with hematoxylin (Sigma) was performed for visualization under the microscope (Aboutaleb et al., 2016). After a washing step, the fluorescence signals from the CA1 area of the right hippocampus fields prepared from each slide were detected with a fluorescence microscope (LABOMED USA, 400× magnification). The number of positive cells and the total number of cells were blindly counted (Image tools software). The results were expressed as the percentage of Bax, Bcl-2, and GFAP-positive cells/total cells.

### 2.6. Statistical analysis

The obtained data are reported as Mean±SD. The Kolmogorov-Smirnov test demonstrated the normality of the distribution. One-way analysis of variance (ANOVA) test was used to compare the differences among the groups. When there was a significant difference, the Scheffe’s or Dunnett’s T3 post hoc test was applied to specify where the difference occurred. When the homogeneity of variance was confirmed, Scheffe’s post hoc test was used; also, we used Dunnett’s T3 post hoc test. The significance level was set at P≤0.05.

## 3. Results

### 3.1. Nesfatin-1 reduced Bax protein levels after cerebral ischemia

**Figure 1.**
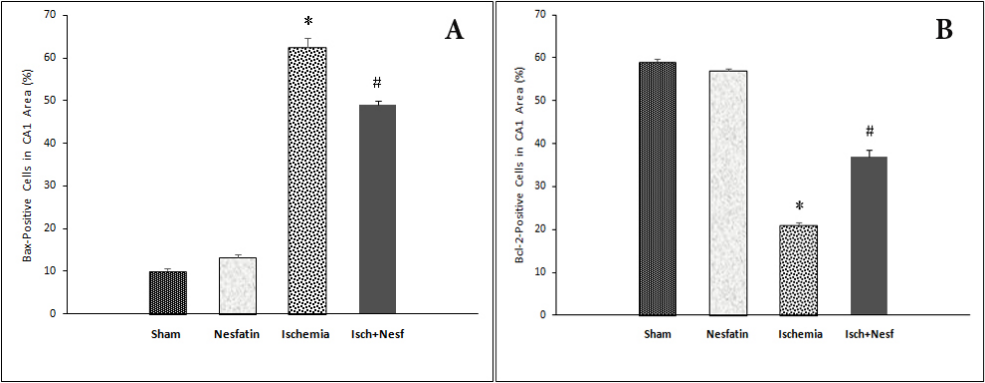
The percentage of Bax-positive cells A. The percentage of active Bax positive cells and B. Active Bcl-2 positive cells in the CA1 area of the hippocampus following cerebral ischemia in different groups * Significantly different compared with sham and nesfatin-1 groups (P<0.001) # Significantly different compared with the ischemia group (P<0.05).

**Figure 2.**
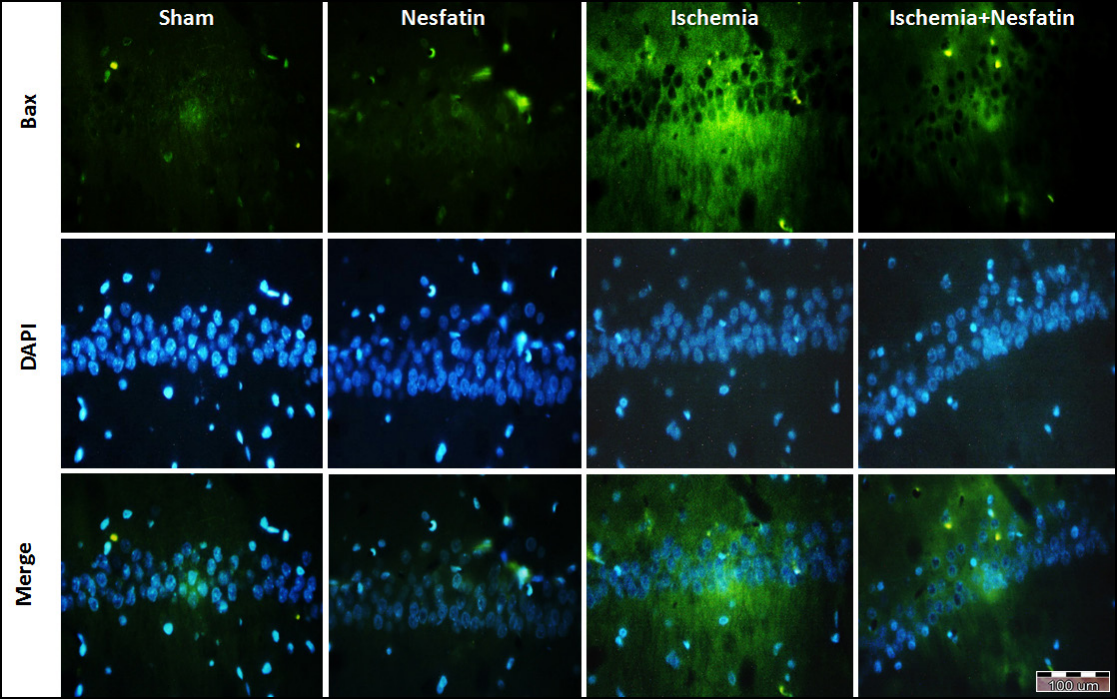
Photomicrographs of immunofluorescence staining of Bax in the hippocampus Immunofluorescence staining was used for identifying Bax activation in the hippocampal CA1 area following the transient cerebral ischemia. Representative Bax-stained (green) and 4′,6-diamidino-2-phenylindole -stained (blue) in the sections (400× magnifications)

The results of Bax immunofluorescence staining showed that in the CA1 area of the hippocampus, there were statistically significant differences among groups with respect to the percentage of Bax-positive cells. Bax was weakly expressed in sham-operated (10.1%±1.24) and nesfatin-1 (13.2%±1.78) groups. Also, the percentage of Bax-positive cells increased in the ischemia group (62.5%±4.74) compared to the sham group (P<0.001). In the nesfatin-1 treatment group, the percentage of Bax-positive cells decreased (49.10%±1.81) compared to the ischemia group (P<0.05) (Figures 1 and 2).

### 3.2. Nesfatin-1 increased Bcl-2 protein level in the CA1 area

**Figure 3.**
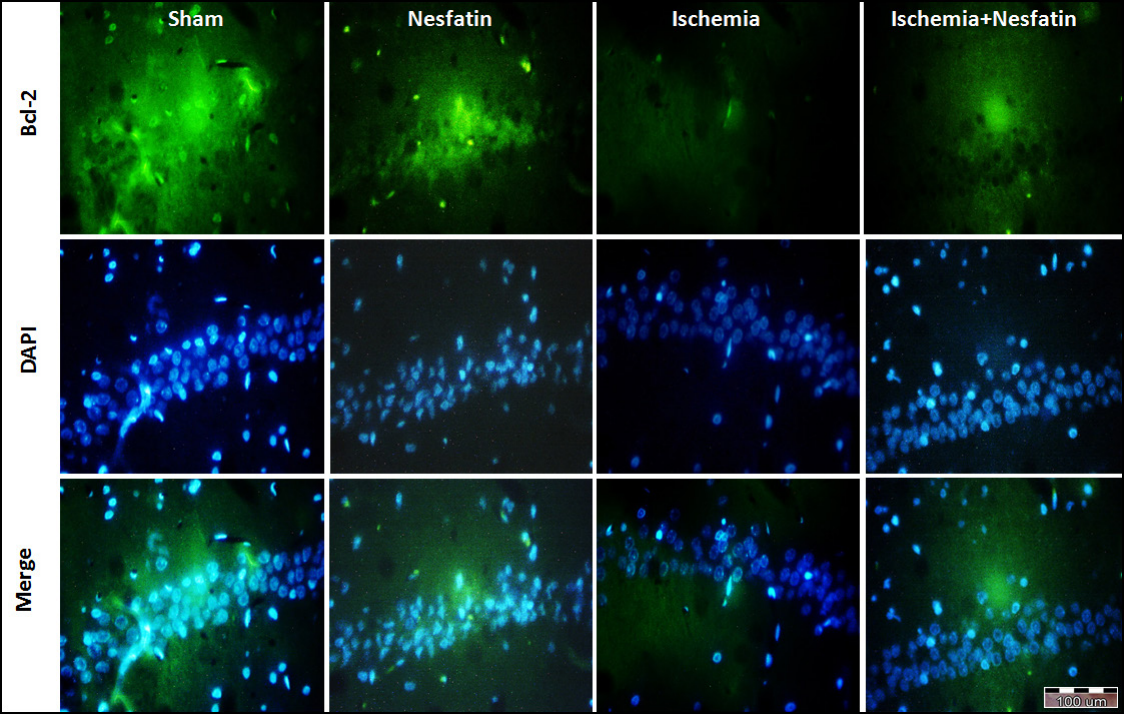
Photomicrographs of immunofluorescence staining of Bcl-2 in the hippocampus Immunofluorescence staining was used for identifying Bcl-2 activation in the hippocampal CA1 area following the transient cerebral ischemia. Representative Bcl-2-stained (green) and 4′,6-diamidino-2-phenylindole-stained (blue) in the sections (400× magnifications)

According to the results of Bcl-2 immunofluorescence staining, there was a significant difference in the percentage of Bcl-2-positive cells among groups. The expression of Bcl-2 protein was higher in the sham (59%±1.58) and nesfatin-1 (57.4%±1.34) groups. The cerebral ischemia decreased the percentage of Bcl-2-positive cells (21.4%±1.55) compared to the sham group (P<0.001). In the nesfatin-1 treatment group, the percentage of Bcl-2-positive cells increased (37.1%±3.31) compared to the ischemia group (P<0.05) (Figures 1 and 3).

### 3.3. Nesfatin-1 reduced the GFAP protein levels after ischemia

**Figure 4.**
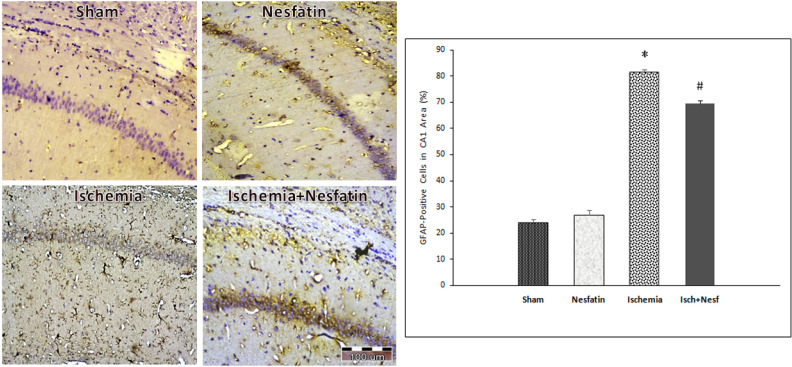
The percentage of glial fibrillary acid protein (GFAP) positive cells in different groups Left: Photomicrographs of immunohistochemical staining of GFAP in the hippocampal CA1 area after transient cerebral ischemia (400× magnifications) Right: Effects of nesfatin-1 on the GFAP levels in the CA1 area following cerebral ischemia * Significantly different compared with sham and nesfatin-1 groups (P<0.001) # Significantly different compared with the ischemia group (P<0.05

The results of GFAP immunohistochemical staining demonstrated a significant difference in the percentage of GFAP-positive cells among groups in the hippocampal CA1 area. GFAP was weakly expressed in the sham group (23.8%±2.94) and nesfatin-1 group (27%±3.8). Additionally, the percentage of GFAP-positive cells increased in the ischemia group (81.4%±2.3) compared to sham and nesfatin-1 groups (P<0.001). In the nesfatin-1 group, the percentage of GFAP-positive cells decreased (69.4%±3.04) compared to the ischemia group (P<0.05) (Figure 4).

## 4. Discussion

This study, for the first time to date, demonstrated the neuroprotective effects of nesfatin-1 against I/R injury by GFAP immunohistochemical staining. So that the number of active GFAP-positive cells significantly increased following transient cerebral ischemia in the hippocampal CA1 area. This evidence suggests that cerebral ischemia causes neuroinflammation intermediary activity of astrocytes in the CA1 area of the hippocampus. On the other hand, treatment with NUCB2/nesfatin-1 considerably reduces the ischemia-reperfusion-induced GFAP expression.

The peptide therapeutic strategy has been applied in the incremental form in recent years. These studies have revealed the peptides’ essential role in the treatment of many diseases, such as infectious and autoimmune diseases (Xiao et al., 2015, Thundimadathil, 2012). On the other hand, the available clinical pharmacological tools to reduce brain injury and treatment for patients with stroke are minimal (Herson & Traystman, 2014). So, it seems that using novel peptides such as nesfatin-1 is a suitable method for the reduction of brain ischemia injury.

Previous studies established that neuroinflammatory mediators play a crucial role in brain ischemia pathophysiology by contribution to ischemic tissue damage (Amantea, Nappi, Bernardi, Bagetta, & Corasaniti, 2009). Reactive astrogliosis accompanies many pathological conditions that affect the central nervous system, such as trauma, neuroinflammation, and ischemic damage. Reactive astrocytes increase the expression of their structural proteins, including GFAP and vimentin (Chen & Swanson, 2003).

Moreover, it was reported that reactive astrocytes following brain ischemia play a vital role in the regulation of inflammation by providing a significant source of the proinflammatory cytokines and chemokines (Morizawa et al., 2017, Kudabayeva et al., 2017, Li et al., 2017). Then, the major pro-inflammatory products, including interleukin-6 (IL-6), interleukin 1 beta (IL-1β), monocyte chemotactic protein-1 (MCP-1/CCL2), and chemokines comprise the cross-talk between activating astrocytes and CNS-infiltrating immune cells, thereby activating infiltrating lymphocytes (Sofroniew, 2015). It was shown that infiltrating leukocytes such as lymphocytes are the main factor of cerebral ischemic inflammation (Li et al., 2017).

One of the possible neuroprotective mechanisms of nesfatin-1 can result from its inflammation suppression properties. Consistent with our observation, the recent studies of Chong-Hui Tang et al. and Derya Özsavcí et al. demonstrated that treatment with nesfatin-1 reduces the concentrations of inflammatory mediators such as interleukin-1 beta, tumor necrosis factor alpha, interleukin-6 after traumatic brain injury, and subarachnoid hemorrhage brain damage (Tang et al., 2012, Özsavcí et al., 2011).

Our results indicate that treatment with novel peptide nesfatin-1 (20 μg/kg) decreases the ischemia-reperfusion-induced pro-apoptotic protein Bax expression and increases the anti-apoptotic protein Bcl-2 expression that was significantly reduced under ischemia conditions.

The pathophysiological mechanisms of cerebral ischemia-reperfusion are complex. Several studies have revealed that reperfusion plays an essential role in the brain ischemia injury and when bloodstream returns to the tissue, it causes a series of processes, including oxidative stress, inflammation, energy failure, excitotoxicity, calcium dysregulation, and the activation of several cell-signaling pathways of neuronal death and apoptosis (Erfani et al., 2015b, Wang C., Liu, M., Pan, Y., Bai, B., & Chen, J., 2017). It seems that any factor that prevents those processes can be used for the treatment of brain ischemia. Cellular apoptosis related to cerebral IR was accompanied by the expression of apoptosis-related genes (Cregan et al., 2002). Besides, several studies showed that the expression of the key antiapoptotic protein Bcl-2 decreased and also the Bax expression as proapoptotic proteins increased at different times of reperfusion (Xing et al., 2008). The correlation between Bcl-2 expression and resistance to apoptosis was observed, resulting from Bcl-2 properties, e.g., Bcl-2 sensitivity to redox changes and antioxidant functions of Bcl-2 during calcium stress attenuates cell death (Doyle, Simon, & Stenzel-Poore, 2008).

The neuroprotection of nesfatin-1 in the CA1 area of the hippocampus after transient global cerebral ischemia can occur by its antiapoptotic and antioxidant activity. Likewise, the recent experiment showed the protective effects of nesfatin-1 in intestinal ischemia-reperfusion by decreasing endothelial nitric oxide syntheses level and oxidative stress index (Ceylan Ayada, Genç, & Akcılar, Şahin, 2015). Moreover the results of Guanjun Jiang et al. study indicate that nesfatin-1 treatment ameliorates acute renal ischemia-reperfusion injury by malondialdehyde level reduction and superoxide dismutase and catalase activities increase. Also, nesfatin-1 lowers apoptotic tubular cell production by a decrease in caspase-3 activity and an increase in the Bcl-2/Bax ratio (Jiang et al., 2015).

The previous observation indicated that transient cerebral ischemia causes the degeneration of vulnerable neurons such as those in the hippocampus, neocortex, and striatum. The neuronal death happened selectively and slowly, which is named “delayed neuronal death” (Hye Kim et al., 2016). Cognitive performances, such as learning and memory, are disrupted following cerebral ischemia. For example, the loss of hippocampal CA1 neurons is related to memory function impairment (Erfani et al., 2015c).

## 5. Conclusion

Because of the importance and sensitivity of CA1 area of hippocampus and the pathophysiological mechanisms of cerebral ischemia, this study provided the novel therapeutic window of nesfatin-1 by the inhibition of astrocytes activation as an inflammatory response, increased the expression of the antiapoptotic protein Bcl-2, and also decrease in Bax-mediated neuronal cell apoptosis after transient cerebral ischemia in rats; however, further experiments are required to clarify these beliefs.

## Ethical Considerations

### Compliance with ethical guidelines

Each animal was used only once. Rats got familiar with their new environment before starting the experimental process. All tests were executed according to the guidelines for the care and use of laboratory animals (National Institutes of Health Newsletter No. 80-23, revised 1996).
